# Melatonin and Caffeine Supplementation Used, Respectively, as Protective and Stimulating Agents in the Cryopreservation of Human Sperm Improves Survival, Viability, and Motility after Thawing compared to Traditional TEST-Yolk Buffer

**DOI:** 10.1155/2019/6472945

**Published:** 2019-10-23

**Authors:** Juliana R. Pariz, Caroline Ranéa, Rosa A. C. Monteiro, Donald P. Evenson, Joël R. Drevet, Jorge Hallak

**Affiliations:** ^1^Androscience, Science and Innovation Center in Andrology and High-Complex Clinical and Andrology Laboratory, São Paulo 04534011, Brazil; ^2^Division of Urology, Department of Surgery, Hospital das Clinicas, University of Sao Paulo Medical School, São Paulo 05403-000, Brazil; ^3^Department of Pathology, Reproductive Toxicology Unit, University of São Paulo Medical School, São Paulo 01246-903, Brazil; ^4^Institute for Advanced Studies, University of Sao Paulo, São Paulo 05508-060, Brazil; ^5^SCSA Diagnostics, Brookings 57006, USA; ^6^Université Clermont Auvergne, GReD Laboratory, Faculty of Medicine, Clermont-Ferrand 63001, France

## Abstract

Cryopreservation processes can damage spermatozoa and impair structural and functional cell characteristics. Plasma, nuclear membranes, and cellular organelles can suffer from the freeze and thaw process. This study evaluates the protective and stimulant effect of melatonin and caffeine supplementation on the functional characteristics of human spermatozoa before and after freezing. Thirty seminal samples from normozoospermic men aged 19–45 years old collected between October 2012 and May 2017 were included. Semen samples were supplemented with either 2 mM melatonin (MEL) prior to cryopreservation, 2 mM caffeine (CAF) in postthaw, or CAF and MEL (CM) in precryopreservation and postthaw, respectively. Kinetics and seminal parameters, mitochondrial activity, DNA fragmentation, and reactive oxygen species (ROS) levels were analyzed before and after cryopreservation. A significant reduction in sperm concentration, total and progressive motility, sperm kinetics, and mitochondrial activity, as well as a significant increase in DNA fragmentation and ROS production in postthaw samples compared to fresh samples, was identified. After administration of a caffeine and/or melatonin supplement, there was a significant increase in progressive motility in the CAF (*p* = 0.005) and CM (*p* = 0.048) groups, as well as mitochondrial activity in the CM group (*p* < 0.05). Cryopreservation has negative effects on overall sperm quality and increases ROS production. A combination of caffeine and melatonin in prefreeze and postthaw sperm samples has proven to be a very effective and simple way to improve semen quality. This will be particularly useful for initial low-quality semen samples, those which suffer the most from the freezing/thawing process.

## 1. Introduction

Sperm cryopreservation is recognized as an essential and relevant aid in the management of infertile patients, regardless of clinical diagnosis. It is of the utmost importance in a variety of clinical situations ranging from cancer to autoimmunity and its immunosuppressive therapeutic strategies to nonmalignant immunological disorders (e.g., lupus erythematous or rheumatoid arthritis) [1–7]. Sperm cryopreservation is also of great help when considering sperm donation and sperm banking, postmortem sperm retrieval, and saving sperm prior to vasectomy; after challenging sperm collection microsurgeries prior to assisted reproduction techniques (ARTs); or in people diagnosed with testicular dysgenesis syndrome or chronically exposed to gonadotoxic substances [4, 8–14].

One of the main disadvantages of sperm cryopreservation is the low postthaw viability, since 25 to 75% of recovered sperm have structural and/or functional damage or do not even survive the mechanical and osmotic stresses associated with the freezing/thawing process, potentially suffering lysis of the sperm cell membrane, specially in morphologically or functionally defective spermatozoa [15, 16]. This is evidenced by a dramatically decreased postthaw motility, which has been associated with higher risk of abortion and congenital malformations after ARTs [17–20]. Besides the deleterious mechanical effects of the freezing/thawing process, most of the detrimental impact of cryopreservation on spermatozoa is linked to oxidative stress [21, 22]. As it is now well-described and accepted by both the clinical and scientific communities, oxidative lesions of spermatozoa have multiple visages affecting sperm structures including plasma membrane, mitochondria, nucleus, and DNA, compromising sperm functions (motility, gamete recognition, and its ability to capacitate as well as its ability to deliver an optimal paternal genetic material into the oocyte). All these consequently strongly affect spermatozoon fertilizing potential and reproductive success and increase the risk of adverse paternally inherited defects in the progeny [23–39]. Therefore, a logical challenge is to attempt to repair/limit some of the damage caused by oxidative stress (OS) during the cryopreservation process in order to maximize gamete survival and function.

Many ingredients have been tested for their ability to reduce OS-mediated damage during cryopreservation of human semen and/or improve sperm mobility after thawing. For example, glutamine, catalase, and ascorbic acid had a protective action and preserved sperm motility when added to cryopreservation media while pentoxifylline supplementation after thawing improved sperm mobility and decreased ROS production [40–43]. Taking advantage of the well-described pleiotropic antioxidant (AO) action of melatonin [44–46] and the well-known stimulating effect of caffeine on sperm motility [47–53], we have decided to evaluate the benefit of complementing our cryopreservation medium with melatonin or our postthaw medium with caffeine or a combination of the two. Thus, the aim of the present study was to investigate the effect of melatonin and caffeine supplementation on the functional characteristics of prefreeze and postthaw human spermatozoa.

## 2. Materials and Methods

### 2.1. Study Design and Patients

This prospective study included 30 normozoospermic samples from male patients aged 22 to 45 years who were treated at Androscience, Science and Innovation Center in Andrology and High-Complex Clinical and Andrology Laboratory, São Paulo, Brazil, from 2013 to 2017. Exclusion criteria included azoospermia, leukocytospermia, necrozoospermia, and other seminal abnormalities such as oligozoospermia < 15 million/ml, progressive motility < 32%, and a percentage of normal morphology <4%. This study was approved by the “Research Ethics Committee” of the University of São Paulo Medical School, Brazil (No. 031/13), and patients gave their informed consent in writing.

**Figure 1:**
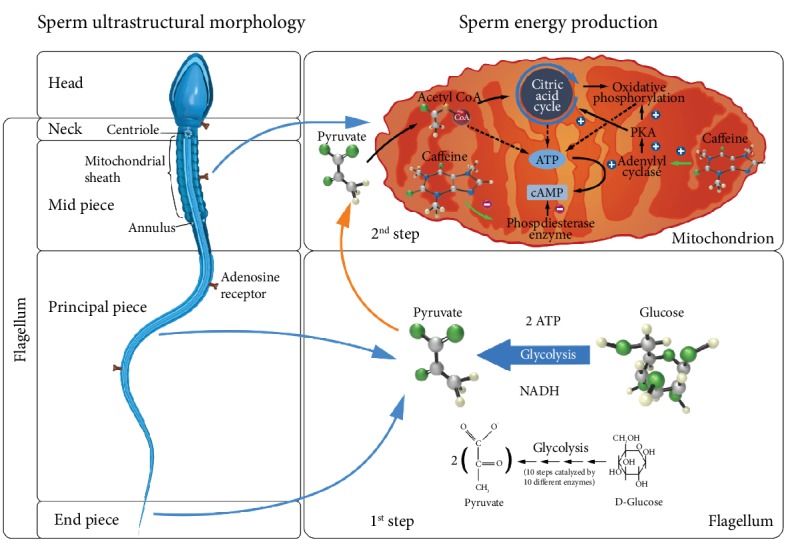
Sperm structure and energy production mechanisms in the sperm cytoplasm and mitochondria. Energy production is initiated by the glycolytic pathway generating two ATPs and one pyruvate molecule. Pyruvate is essential for the initiation of the acid citric cycle and the oxidative phosphorylation steps of the mitochondrial pathway to produce ATP. Caffeine binds to the adenosine receptor to stimulate the adenylyl cyclase, an enzyme present in mitochondria, leading to the conversion of ATP into cAMP. Protein kinase A (PKA) is activated by cAMP which increases cytochrome c oxidase (COX) activity and oxidative phosphorylation. Caffeine also acts as an inhibitor of phosphodiesterase enzyme activity, inhibiting the degradation of cAMP and promoting an increase in its intracellular concentration.

The samples were subdivided into two groups and cryopreserved by the slow freezing technique using modified human tubal fluid (modified HTF, Irvine Scientific, Santa Ana, CA, USA) without supplementation or with 2 mM melatonin (Sigma-Aldrich, Saint Louis, MO, USA). After thawing, each group was then subdivided into two samples, one of which was supplemented with 2 mM caffeine (1,3,7-trimethylxanthine; Sigma-Aldrich). The respective concentrations of MEL and CAF used in the course of this study were determined following a survey of the available literature. For MEL, it corresponds to the circulating human concentration. For CAF, we worked out the optimal concentration to be used in an earlier study [47] which roughly corresponds to a typical circulating concentration after the ingestion of one cup of coffee. Therefore, five groups were generated (see Figure 1): prefrozen samples; postthaw control samples without supplementation (CONT); postthaw samples with either melatonin (MEL), added before cryopreservation, or caffeine (CAF), used after thawing; or both additives: melatonin+caffeine (MC).

### 2.2. Semen Analysis and Sperm Functional Tests

Semen analysis and functional tests were performed on all precryopreservation and postthaw samples. Semen samples were collected by masturbation after a period of 2 to 5 days of ejaculatory abstinence. Sperm counts were made manually using a Makler counting chamber (Sefi Medical Instruments, Haifa, Israel), and concentrations are expressed in million cells/ml. Sperm kinetic parameters including progressive (%PR) and total motility (%TM) [54], curvilinear velocity (VCL; *μ*m/s), linear velocity (VSL; *μ*m/s), mean velocity (VAP; *μ*m/s), linearity (LIN; %), straightness (STR; %), wobble (WOB; %), and hyperactivity (HYP; %) were measured via the semiautomated platform “Sperm Class Analyzer” (SCA, MICROPTIC, Barcelona, Spain).

Mitochondrial activity was assessed by staining with 3,3′-diaminobenzidine (DAB). Fifty *μ*l of semen was added to a solution containing 1 mg/ml of DAB (Sigma-Aldrich) in an equal volume of PBS (137 mM NaCl, 2.7 mM KCl, 4.3 mM Na_2_HPO_4_, 1.4 mM KH_2_PO_4_, pH 7.4; Sigma-Aldrich) and incubated in the dark for 1 hour at 36.7°C. Two 10 *μ*l smears were prepared on microscope slides and fixed in 10% formaldehyde for 10 minutes. About 200 sperm were observed under a phase contrast microscope (×100 oil-immersed objective—Eclipse E200, Nikon, Tokyo, Japan). Each sperm was classified as follows: Class I (DAB I; 100% of the colored centerpiece, with a prominent colored reaction showing the mitochondrial sheath as a compact cylinder), Class II (DAB II; more than 50% of the centerpiece colored but in a dispersed/patchy fashion), Class III (DAB III; less than 50% of the centerpiece colored), and Class IV (DAB IV; no coloration in the centerpiece) [55].

ROS levels were analyzed by light emission after stimulation with a luminal chemiluminescent probe [56, 57]. One aliquot of each sample (400 *μ*l) was added to 20 *μ*l of a luminol solution containing 5 mM luminol added to DMSO (Sigma-Aldrich) and evaluated using a luminometer (Berthold Technologies, Bad Wildbad, Germany). The results are expressed in 10^4^ photons counted per minute (cpm) for 20 × 10^6^ spermatozoa [56, 57]. Evaluation of sperm DNA integrity was performed using the SCSA (Sperm Chromatin Structure Assay) method [58]. An aliquot fraction of the semen was diluted in a TNE buffer (0.15 M NaCl, 0.01 M Tris-HCl, 1 mM EDTA, ph = 7.4; Sigma-Aldrich) at a concentration of 1-2.10^6^ sperm/ml. An acid detergent solution (400 *μ*l; 0.08 M HCl, 0.15 M NaCl, and 0.1% Triton X-100 pH 1.2; Sigma-Aldrich) was then added to the samples. The cells were then stained with 1.2 ml of acridine orange solution (0.1 M citric acid, 0.2 M Na_2_HPO_4_, 1 mM EDTA, and 0.15 M NaCl, pH 6.0; Sigma-Aldrich) containing 6 *μ*g/ml acridine orange (Polysciences Inc., Washington, PA, USA). Five thousand sperm cells were evaluated using a flow cytometer (BD Biosciences, San Jose, CA, USA). Data was analyzed using SCSA software to determine the DNA fragmentation index (DFI%).

### 2.3. Sperm Cryopreservation

Seminal samples were subdivided into two aliquots of 1 ml each and processed using the Isolate method (Irvine Scientific). One ml two-layer density gradient (40%/80%; ISolate, Irvine Scientific) were constructed onto which were loaded 1 ml of seminal samples. Samples were centrifuged at 400g for 15 minutes at 36.6°C and supernatants carefully aspirated. One sperm pellet was resuspended in 1 ml of modified-HTF medium, and melatonin (2 mM in modified HTF) was added to the other pellet.

Cryopreservation by slow freezing cryopreservation initiates by addition of one volume of a glycerol-based cryoprotectant TEST-yolk buffer (TYB; Irvine Scientific) to each sample. All samples were subjected to the following sequential protocol: 8 min at -20°C, 1 hour in nitrogen vapor (-79°C), and finally immersion in liquid nitrogen (-196°C) for extended storage [59].

The thawing process included samples incubated at 25°C for 15 minutes and then at 36.7°C for 15 minutes [60]. After complete thawing, each sample was separated into two aliquots. Cryoprotectant solution was removed by centrifugation (400g for 10 minutes), and sperm pellet was resuspended in either HTF-modified or 2 mM solution of caffeine in modified HTF. Both samples were then incubated at 36.7°C for an additional 15 minutes [47, 61].

### 2.4. Statistical Analysis

The normality of semen parameters was verified and evaluated using the Kolmogorov-Smirnov test. The Student paired *t*-test was chosen to compare data between two groups (prefrozen samples and CONT). ANOVA with analysis of repeated measurements and the Holm-Sidak post hoc test were used to compare CONT with the CAF, MEL, and CM groups. In addition, an interaction test was performed to evaluate synergistic interactions and the combined effects of caffeine and melatonin supplementation. All analyses were performed using the SPSS version 19.0, and a value of *p* < 0.05 was considered statistically significant.

## 3. Results

Thirty normozoospermic seminal samples from patients aged 22 to 45 years were included in this study (mean age = 33.74 ± 7.36 years). Patients were referred for a variety of andrological reasons, including fertility assessment (50.0%), prevasectomy evaluation (16.7%), infertility (10.0%), premature ejaculation (6.7%), erectile dysfunction (6.7%), testicular pain (3.3%), postvasectomy reversal (3.3%), and varicocele (3.3%).

### 3.1. Initial Seminal Analysis after Freezing/Thawing

The main parameters of the precryopreservation (fresh) and postthaw samples without supplementation (CONT) were analyzed (Table 1). The classic negative effects of cryopreservation on sperm motility were clearly observed. In particular, we recorded a significant decrease in progressive and total motility in postthaw sperm samples (*p* < 0.001). Thanks to the detailed SCA analysis, we recorded a decrease in almost all kinetic parameters including VCL, VSL, VAP, LIN, STR, and WOB (*p* < 0.009) with the exception of hyperactivity (HYP; *p* = 0.073). In addition, a significant reduction in mitochondrial activity after thawing was observed in the DAB I and DAB II groups (*p* < 0.001). Finally, as expected too, semen samples after thawing showed an increase in ROS levels (*p* = 0.052, with important increase in postthaw samples and biological relevance) and DFI% (*p* = 0.046) when compared to the precryopreservation (CONT) group.

### 3.2. Melatonin and Caffeine Supplementation

As shown in Table 2, there was a significant increase in progressive motility (*p* < 0.05) in the CAF and MC samples compared to the CONT samples. However, no statistically significant improvement was recorded by examining each specific kinetic parameter individually via the SCA platform. Combined supplementation (CM) resulted in an increase in mitochondrial activity, as evidenced by the higher proportion of DAB I sperm (*p* < 0.001) and the concomitant decrease in the proportion of DAB IV sperm (*p* < 0.001). No statistically significant effects were observed on DFI% and ROS concentrations under the tested conditions.

## 4. Discussion

This study evaluated the effect of MEL and CAF addition on the functional characteristics of normozoospermic sperm samples before cryopreservation and postthawing, using the slow freezing method and glycerol as a cryoprotectant.

As expected, the cryopreservation process has had its panel of associated adverse effects reducing sperm motility and mitochondrial activity while increasing sperm ROS levels and DNA fragmentation [15, 17, 27, 39, 62–68]. In the present study, sperm motility went down from 50.22% in fresh semen to 7.5% postthawing. Also, a decrease in total sperm count was expected due to the known severity of the cryopreservation-thawing processes and increased sperm cell membrane fragility in defective spermatozoa submitted also to seminal processing with further loss due to cell lysis [16]. Most individual sperm velocity parameters (VCL, VAP, LIN, STR, and WOB) were significantly decreased after cryopreservation in total agreement with earlier reports (as an example, see [17]), a situation that has been associated with the low success rate of ART procedures such as IUI (intrauterine insemination) when cryopreserved sperm samples are used [67]. In addition, completing the classical picture, sperm mitochondria activity was significantly reduced after cryopreservation and was associated with an increase in ROS and its associated effect (i.e., higher DNA fragmentation index) revealing alteration in the integrity of the sperm nucleus [21, 27, 62, 63, 68].

**Figure 2:**
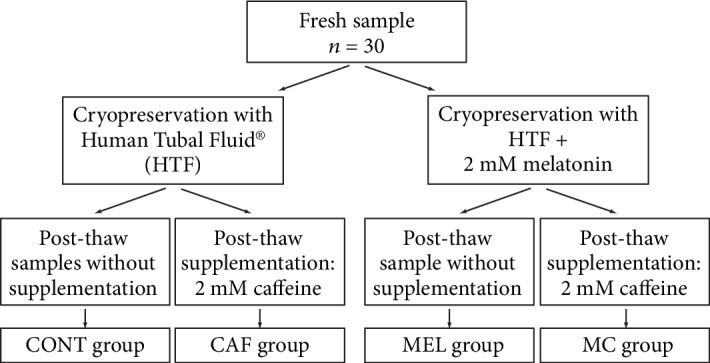
Study design. CONT group: control (no supplementation) group; CAF group: caffeine treatment group; MEL group: melatonin treatment group; MC group: melatonin+caffeine treatment group.

The addition of CAF during thawing resulted in a significant increase (almost twice as high) in the percentage of progressive motile sperm, a situation that was slightly reinforced when MEL was also added to the cryopreservation step without providing a very convincing additive/synergistic effect. It is interesting to note that when both additives were used, a significant improvement in sperm mitochondrial activity was recorded, whereas this was not the case when the additives were used separately. This result may suggest a synergistic action of MEL and CAF in protecting and stimulating, respectively, the structure and function of mitochondria. In fact, CAF can act as an inhibitor of the enzymatic activity of phosphodiesterase, which is responsible for the degradation of cyclic adenosine monophosphate (cAMP) resulting in an increase in intracellular cAMP concentration. CAF can also act as a protein kinase A (PKA) stimulator by enhancing cytochrome c oxidase (COX) activity and oxidative phosphorylation (Figure 2) [47–50, 53]. In addition, the protective action of MEL on sperm mitochondria may explain the lower levels of ROS recorded in the different samples tested (although this was just a trend). Melatonin is a known powerful antioxidant that also plays a role in stimulating mitochondrial respiration and ATP synthesis, as well as increasing the activity of respiratory chain complexes I and IV (oxidative phosphorylation) [45]. Thus, the protective action of MEL on sperm can also explain the higher concentrations of viable sperm after thawing recorded in MEL and MC samples (although again a strong statistical significance was not achieved) ultimately preventing sperm from passing into the apoptotic pathway [41, 51, 53].

Our data are in agreement with an earlier study that has shown in 43 human semen samples that melatonin addition resulted in an increase in sperm motility and vitality and in reduced ROS generation and lipid peroxidation [69]. They are also in line with *in vitro* studies in large domestic animals that have shown that treatment of semen samples with melatonin significantly increased sperm motility [70, 71], reduced sperm membrane lipid peroxidation [24, 72], prevented sperm capacitation and apoptosis [73], and protected spermatozoa against high levels of ROS [51]. Our data also concur with the observation that men with higher levels of MEL (or its downstream metabolites) have better seminal parameters including sperm concentration, motility, and normal morphology [71]. It also makes sense with the observation that infertile men exhibit lower levels of MEL when compared to fertile ones [71].

Regarding CAF addition to postthaw sperm samples, reports are scarce and mostly concern large domestic animals for which IUI with frozen sperm is commonly used. As we report in this study, it was shown that addition of CAF to postthaw bull sperm resulted in an increase in sperm motility among other parameters that were also ameliorated including sperm capacitation and acrosome reaction, leading to better gestation rates [74, 75]. In humans, the effect of CAF was mainly evaluated on spermatogenesis resulting in the observation that high CAF intake (>800 mg/day) was associated with reduced sperm concentration [50]. One study conducted with fertile men did show that the consumption of more than 6 coffee cups per day resulted in an increase in sperm motility [48, 76]. As energy expenditure is one of the major factors involved in the loss of fertilization capacity after sperm cryopreservation [77], the potential CAF-mediated increase in intracellular energy production should be investigated further.

## 5. Conclusions

Although preliminary and perfectible, this study illustrates that there is room for improvement of the postthaw performance of frozen human semen samples via the addition of a protectant or/and stimulant. This could prove to be useful with seminal samples of poor initial quality whatever the cause. In our example, CAF or CAF+MEL (MC) resulted in an improvement of postthaw sperm motility. In addition, and solely when CAF+MEL was used, sperm motility improvement was associated with a healthier mitochondrial status. In practice, these simple actions could logically translate into higher ART success rate, an issue that remains to be evaluated.

## Figures and Tables

**Table 1:** Mean, standard deviation (SD), and minimum and maximum (min–max) values in precryopreservation and postthaw sperm samples without supplementation.

	Pre-cryopreservation *N* = 30	Postthaw *N* = 30	*p* value
Concentration (million/ml)			
Mean; SD	62.48; 47.32	18.13; 11.94	**<0.001**
Min–max	22.00-240.00	1.90-45.00	
Progressive motility (%)			
Mean; SD	50.22; 10.96	7.50; 2.71	**<0.001**
Min–max	35.00-75.00	0.00-50.00	
Total motility (%)			
Mean; SD	66.09; 8.94	16.13; 12.27	**<0.001**
Min–max	50.00-90.00	0.00-50.00	
Curvilinear velocity (VCL) (*μ*m/s)			
Mean; SD	55.69; 10.89	29.47; 20.27	**<0.001**
Min–max	5.80-69.00	0.00-64.33	
Straight-line velocity (VSL) (*μ*m/s)			
Mean; SD	19.45; 7.70	7.61; 8.97	**0.002**
Min–max	11.58-40.14	0.00-32.58	
Average path velocity (VAP) (*μ*m/s)			
Mean; SD	30.66; 8.12	14.12; 11.43	**<0.001**
Min–max	0.00-37.38	24.23-58.46	
Linearity (LIN) (%)			
Mean; SD	34.54; 9.53	20.34; 15.01	**0.005**
Min–max	24.23-58.46	0.00-63.19	
Straightness (STR) (%)			
Mean; SD	62.27; 9.50	21.86; 18.63	**<0.001**
Min–max	50.45-82.38	0.00-67.61	
Wobble (WOB) (%)			
Mean; SD	54.70; 6.66	41.90; 15.62	**0.009**
Min–max	47.62-70.96	0.00-72.50	
Hyperactivity (%)			
Mean; SD	7.97; 7.46	2.47; 6.83	0.073
Min–max	0.90-25.75	0.00-25.00	
DNA fragmentation index (%)			
Mean; SD	36.86; 15.29	47.93; 18.09	**0.046**
Min–max	12.00-64.00	11.00-92.00	
DAB I (%)			
Mean; SD	26.19; 15.61	9.43; 7.23	**<0.001**
Min–max	4.00-66.00	0.00-23.00	
DAB II (%)			
Mean; SD	47.62; 17.39	51.48; 12.18	0.418
Min–max	3.00-24.00	28.00-71.00	
DAB III (%)			
Mean; SD	11.67; 5.54	21.19; 7.84	**<0.001**
Min–max	6.00-44.00	3.00-24.00	
DAB IV (%)			
Mean; SD	12.95; 8.92	18.04; 10.94	0.106
Min–max	2.00-38.00	3.00-42.00	
ROS level (10^4^ cpm/20 × 10^6^ sperm)			
Mean; SD	0.92; 0.76	3.22; 4.94	0.052^∗^
Min–max	0.20-3.11	0.03-19.53	

DAB: mitochondrial activity grade; ROS: radical oxygen species. Values in bold are statistically significant (*p* < 0.05) based on Student's paired *t*-test. ^∗^*p* slightly >0.05 not statistically significant but is biologically relevant.

**Table 2:** Mean, standard deviation (SD), and minimum and maximum (min–max) values in postthaw samples without supplementation (CONT), and postthaw samples supplemented with melatonin (MEL), caffeine (CAF), or melatonin+caffeine (MC).

	CONT *N* = 30	CAF *N* = 30	MEL *N* = 30	CM *N* = 30	*p* value		
					CONT vs. CAF	CONT vs. MEL	CONT vs. CM
Concentration (million/ml)							
Mean; SD	18.13; 11.94	18.19; 10.82	21.05; 14.83	21.62; 14.90	0.662	0.497	0.166
Min–max	1.90-45.00	4.00-39.00	5.00-69.00	5.00-67.00			
Progressive motility (%)							
Mean; SD	7.50; 2.71	13.27; 2.62	9.49; 8.28	16.54; 13.25	**0.005**	0.980	**0.048**
Min–max	0.00-50.00	0.00-50.00	0.00-23.70	0.00-44.00			
Total motility (%)							
Mean; SD	16.13; 12.27	20.24; 13.02	19.94; 11.84	24.80; 15.08	0.466	0.813	0.078
Min–max	0.00-50.00	0.00-50.00	5.00-40.00	0.00-50.00			
Curvilinear velocity (VCL)(*μ*m/s)							
Mean; SD	29.47; 20.27	41.53; 20.71	37.81; 18.43	46.38; 21.87	0.999	1.000	0.626
Min–max	0.00-64.33	15.33-109.11	15.49-60.45	13.84-97.63			
Straight-line velocity (VSL) (*μ*m/s)							
Mean; SD	7.61; 8.97	10.67; 6.16	7.80; 5.48	16.62; 19.03	1.000	0.961	0.729
Min–max	0.00-32.58	0.82-21.43	2.69-14.82	0.70-58.22			
Average path velocity (VAP) (*μ*m/s)							
Mean; SD	14.12; 11.43	19.47; 10.30	16.85; 10.14	26.09; 18.09	1.000	0.989	0.462
Min–max	24.23-58.46	2.47-49.14	7.20-29.33	1.55-59.23			
Linearity (LIN) (%)							
Mean; SD	20.34; 15.01	24.44; 5.00	18.51; 5.16	30.81; 23.28	1.000	0.899	0.693
Min–max	0.00-63.19	5.36-29.92	12.71-26.48	5.09-83.11			
Straightness (STR) (%)							
Mean; SD	21.86; 18.63	52.69; 7.04	43.45; 6.28	54.38; 19.85	1.000	0.917	0.926
Min–max	0.00-67.61	33.33-59.34	36.74-56.31	40.31-98.30			
Wobble (WOB) (%)							
Mean; SD	41.90; 15.62	45.68; 5.54	42.17; 6.37	52.25; 14.85	0.946	0.866	0.669
Min–max	0.00-72.50	16.08-52.87	34.61-49.23	11.18-84.55			
Hyperactivity (%)							
Mean; SD	2.47; 6.83	2.37; 1.80	0.78; 0.90	1.23; 1.61	0.995	0.929	0.966
Min–max	0.00-25.00	0.00-5.36	0.00-2.41	0.00-9.09			
DNA fragmentation (%)							
Mean; SD	47.93; 18.09	43.67; 18.00	45.07; 22.56	44.13; 23.26	0.901	0.995	0.988
Min–max	11.00-92.00	9.00-96.00	10.00-97.00	11.00-97.00			
DAB I (%)							
Mean; SD	9.43; 7.23	13.18; 10.35	11.68; 10.60	19.14; 13.44	0.155	0.526	**0.001**
Min–max	0.00-23.00	0.00-36.00	0.00-42.00	3.00-48.00			
DAB II (%)							
Mean; SD	51.48; 12.18	52.82; 11.07	51.45; 13.65	53.23; 12.88	0.902	1.000	0.945
Min–max	28.00-71.00	31.00-72.00	7.00-69.00	20.00-73.00			
DAB III (%)							
Mean; SD	21.19; 7.84	19.45; 8.03	19.54; 10.74	17.77; 6.62	0.846	0.967	0.230
Min–max	3.00-24.00	2.00-35.00	8.00-63.00	5.00-33.00			
DAB IV (%)							
Mean; SD	18.04; 10.94	16.36; 10.26	17.31; 5.59	10.00; 5.51	0.580	0.489	**<0.001**
Min–max	3.00-42.00	2.00-45.00	0.00-34.00	4.00-24.00			
ROS level (10^4^ cpm/20 × 10^6^ sperm)							
Mean; SD	3.22; 4.94	2.95; 4.43	1.81; 2.17	2.52; 2.57	1.00	0.849	0.998
Min–max	0.03-19.53	0.02-17.95	0.00-12.73	0.00-9.74			

DAB: mitochondrial activity grade; ROS: radical oxygen species. Values in bold are statistically significant (*p* < 0.05) based on ANOVA and the Holm-Sidak post hoc test.

## Data Availability

The data used to support the findings of this study are available from the corresponding author upon request.
